# The Cycling Brain in the Workplace: Does Workload Modulate the Menstrual Cycle Effect on Cognition?

**DOI:** 10.3389/fnbeh.2022.856276

**Published:** 2022-06-02

**Authors:** Min Xu, Dandan Chen, Hai Li, Hongzhi Wang, Li-Zhuang Yang

**Affiliations:** ^1^Bengbu Medical College, Bengbu, China; ^2^Hefei Cancer Hospital, Chinese Academy of Sciences, Hefei, China; ^3^Anhui Province Key Laboratory of Medical Physics and Technology, Institute of Health and Medical Technology, Hefei Institutes of Physical Science, Chinese Academy of Sciences, Hefei, China

**Keywords:** menstrual cycle, workload, cognitive flexibility, inhibitory control, divided attention

## Abstract

Recent decades have witnessed increased research efforts to clarify how the menstrual cycle influence females’ cognitive and emotional functions. Despite noticeable progress, the research field faces the challenges of inconsistency and low generalizability of research findings. Females of reproductive ages are a heterogeneous population. Generalizing the results of female undergraduates to women in the workplace might be problematic. Furthermore, the critical cognitive processes for daily life and work deserve additional research efforts for improved ecological validity. Thus, this study investigates cognitive performance across the menstrual cycle using a sample of young nurses with similar duties. We developed a mini-computerized cognitive battery to assess four mental skills critical for nursing work: cognitive flexibility, divided attention, response inhibition, and working memory. Participants completed the cognitive battery at menses, late-follicular, and mid-luteal phases. In addition, they were classified into low- and high workload groups according to their subjective workload ratings. Our results demonstrate a general mid-luteal cognitive advantage. Besides, this study reveals preliminary evidence that workload modulates the menstrual cycle effect on cognition. Only females of low workload manifest the mid-luteal cognitive advantage on divided attention and response inhibition, implying that a suitable workload threshold might be necessary for regular neuro-steroid interactions. Thus, this study advocates the significance of research focusing on the cycling brain under workloads.

## Introduction

Ovarian hormones, such as estradiol and progesterone, fluctuate during the menstrual cycle in healthy females of reproductive age. The estradiol levels gradually increase after the menses phase, peaking in the late follicular phase and then dropping after ovulation and rising again in the mid-luteal phase to moderate levels. The progesterone levels increase after ovulation and peak in the middle of the luteal phase. Then, the two ovarian hormones drop to the lowest levels before the onset of the next menses. Thus, the menstrual cycle is a convenient and ecological model of ovarian hormones. Recent years have witnessed an explosion of research on how sex hormones and the menstrual cycle shape female brains (Barth et al., 2015; Ycaza Herrera et al., 2019; Beltz and Moser, 2020; Le et al., 2020; Dubol et al., 2021; Hidalgo-Lopez and Pletzer, 2021).

Estradiol and progesterone have neuroactive effects. The hypothalamic-pituitary-gonadal (HPG) axis regulates the reproductive processes and modulates cognitive and emotional functions through direct or indirect projection to the prefrontal cortex, hippocampus, thalamus, and brainstem (Morrison et al., 2006; Le et al., 2020). It has long been hypothesized that cognitive performance across the menstrual cycle might vary due to the fluctuation of ovarian hormones. The effect of the menstrual cycle has been found on social preference (Durante et al., 2014; Zhuang and Wang, 2014; Wang and Chen, 2020; Wang et al., 2021), cognitive ability (Hussain et al., 2016; Hidalgo-Lopez and Pletzer, 2017; Leeners et al., 2017; Pletzer et al., 2017; Scheuringer and Pletzer, 2017), motor learning (Ikarashi et al., 2020), cortical structures (Lisofsky et al., 2015; Catenaccio et al., 2016; Pletzer et al., 2018), and brain functions (Barth et al., 2016; Diekhof and Ratnayake, 2016; Hidalgo-Lopez and Pletzer, 2019, 2021; Pletzer et al., 2019; Wang et al., 2020a). Moreover, the late follicular or luteal phase advantage on cognition has always been advocated because of high neuroprotective steroids (Sundstrom-Poromaa and Gingnell, 2014; Zhuang et al., 2020; Wang et al., 2021). For example, previous studies suggest that females show superior social cognitive performance during their luteal than menses or follicular phase (Wang and Chen, 2020; Wang et al., 2020a,b, 2021). Although the neuroprotective role of estradiol has been advocated, the role of progesterone remains ambiguous (Baudry et al., 2013). Some studies suggest that progesterone may antagonize rather than synergize estradiol effects (Rosario et al., 2006; Carroll et al., 2008). It is hard to separate the effects of progesterone and estradiol across the menstrual cycle because both hormone levels are high in the mid-luteal phase. Thus, studying the three different phases across the menstrual cycle is necessary to clarify the relationship between hormones.

Despite the appealing association between the menstrual cycle and cognition, the empirical evidence is far from consistent (Sundstrom-Poromaa and Gingnell, 2014; Sundstrom-Poromaa, 2018; Beltz and Moser, 2020; Le et al., 2020). The evolutionary hypothesis implicated that women might show visuospatial ability advantage during their early follicular phase (low ovarian steroids) and verbal ability advantage in the luteal phase (high ovarian steroids). However, Sundstrom-Poromaa and Gingnell (2014) summarized that the supporting evidence is insufficient in the literature. In addition, their following review further suggests that the menstrual cycle might influence emotion, but has a limited effect on cognitive function (Sundstrom-Poromaa, 2018). However, recent neuroimaging studies have revealed consistent evidence that the menstrual cycle modulates the structure, functional activation, and connectivity of brain regions that are responsible for cognitive control (e.g., prefrontal cortex) and memory (e.g., hippocampus) (Beltz and Moser, 2020; Dubol et al., 2021). Thus, it might be too soon to reject the menstrual cycle’s potential effect on “cold” cognition.

The inconsistency might be because the effect of the menstrual cycle is too transient to be captured by behavioral assessment or noise due to methodological flaws (Le et al., 2020). However, it has been long overlooked in the field that healthy females of reproductive age are a heterogeneous population with huge variability in their social-economic status, years of education, social support, occupation, and work pressure. Many studies recruited undergraduate or graduated female students from the campus or females in nearby communities of different professions. These findings might not generalize seamlessly to some specific populations. Only a few studies have employed homogenous samples within a particular workplace, such as nurses (Hatta and Nagaya, 2009). Investigating the cycling brain in specific workplaces is, thus, a valuable research direction.

Furthermore, the inconsistency might be due to the menstrual cycle’s interaction with other factors (Bernal and Paolieri, 2022). It has been proposed that estrogen and progesterone interact with cognition-related neurotransmitter systems, including serotoninergic, dopaminergic, gamma-aminobutyric-acid (GABA)-ergic, and glutamatergic pathways, with profound effects on brain structure and function (Barth et al., 2015). For example, recent evidence shows that the effect of estradiol status on working memory function depends on the baseline dopamine levels (Jacobs and D’Esposito, 2011). Using the eye blink rate (EBR), an indicator of striatal dopamine levels, one following study reveals that females with lower EBR showed superior Stroop performance during their luteal phase and vice versa (Hidalgo-Lopez and Pletzer, 2017). Hidalgo-Lopez and Pletzer (2019) recent work also suggests that baseline performance modulates the menstrual cycle effect on the inhibitory control ability. Besides the factors mentioned above, there might be many contextual and individual factors deserving increasing research attention.

Female nurses account for 90% of the global nursing workforce and play irreplaceable roles in public health (World Health Organization, 2020). Meanwhile, female nurses undertake noticeable workloads. For example, in China, on average, a nurse in a general hospital takes care of 8 patients in the daytime and 23 patients at night (Shen et al., 2020). Compared with other careers, the nursing job characterizes by mental pressures induced by multitasking and attentional interferences. Potter et al. (2005) use a cognitive task analysis methodology to reveal that a nurse must hold 11 activities in mind in the acute care work setting. The nursing job is also full of interruptions associated with procedure failures and clinical errors (Westbrook et al., 2010). Thus, an efficient nurse needs to switch flexibly among tasks (cognitive flexibility), attend to patients and clinical signals simultaneously (divided attention), inhibit automatic, habitual but inappropriate actions (response inhibition), and store necessary information in mind (working memory). Research focusing on the female nurse population is, thus, valuable for promoting their occupational health.

Previous studies have suggested that hormones from the hypothalamic-pituitary-adrenal (HPA) axis regulate the HPG axis (Oyola and Handa, 2017). The HPA axis is the coordinator of the brain’s fight-or-flight response, which increases cortisol production to deal with stressful events. Previous studies have also demonstrated an inverted U-shaped relationship between workload and task performance (Ma et al., 2020). However, it is still ambiguous whether workload would interact with the menstrual cycle to affect cognitive performance. This study investigates whether workload modulates the cycling brain using a homogenous nurse sample. The workload here refers to the cognitive, emotional, and physiological resources expended to complete the task requirement (Alghamdi, 2016). We chose four representative cognitive paradigms (task-switching, divided attention, spatial Stroop, and multiple change detection) to target core mental skills necessary for nursing work. In addition, a self-report measure, namely the National Aeronautics and Space Administration Task Load Index (NASA-TLX), quantifies the nursing workload, which can tease apart six sources of work pressures (Hart, 2016). Although the workload can be evaluated physiologically (Borghini et al., 2014), self-report measures are helpful to provide a convenient, inexpensive, reliable, and valid sampling (Wickens, 2008). We hypothesized that female nurses perform better during their mid-luteal phase. In addition, workload might be a potential modulatory factor of the menstrual cycle effect.

## Materials and Methods

### Participants

We recruited 96 healthy right-handed female registered nurses in a local hospital. All of them had a regular menstrual cycle of 24–35 days (Le et al., 2020) and variability between cycles of less than 7 days in the past 3 months, with normal or corrected-to-normal vision, had not taken oral contraceptive or other hormonal medications within the previous 3 months, no history of nicotine or alcohol abuse, no sleep disorders, and no neurological, psychiatric, or endocrine disorders, including premenstrual dysphoric disorder (PMDD) and premenstrual syndrome (PMS). Ten participants were excluded due to their actual cycle phase falling out of the normal range during the experiment session according to their follow-up report on the onset of the next cycle. Seven participants dropped out for personal reasons, leaving a final sample of 79 nurses (*M* = 25.52 years, SD = 4.33 years) with a mean cycle length of 29.42 days (SD = 1.69). The study was approved by the local ethics committee and was conducted following the Declaration of Helsinki. All participants gave written informed consent and received monetary compensation.

### Research Procedure

Participants completed an online screening questionnaire to determine whether they were eligible to participate in the study. Eligible participants enrolled in the test session were required to record their menses’ start date and duration for at least 3 months to double-check whether their menstrual cycle is regular. The first author (MX) interviewed them privately to survey their subjective nursing workload and check their menstrual cycle information and calculate their cycle phase for those participants. The menstrual cycle mapping was determined using the backward counting procedure widely used in the literature (Zhuang and Wang, 2014; Hidalgo-Lopez and Pletzer, 2017; Schaumberg et al., 2017; Wang et al., 2021). Specifically, we defined the menses phase (low estradiol levels, low progesterone levels) as the 1–4 days after the onset of menstruation; the late-follicular phase (estradiol levels peak and low progesterone levels) as the 3 days before the predicted ovulation; and the mid-luteal phase (moderate estradiol levels and high progesterone levels) as 3 days after the expected ovulation to 3 days before the next onset of the menstruation. We calculated the predicted ovulation by subtracting 14 days from the expected next menstruation onset, determined using each participant’s average cycle length in the last 3 months.

The study was a within-subject, longitudinal design. Thus, participants attended three behavior test sessions during the menses, late-follicular, and mid-luteal phases. These cycle phases were set apart by at least 6 days. The starting session was counterbalanced among participants. About one-third of participants started their first session in the menses, one-third in the late-follicular, and one-third in the mid-luteal phase. For each test session, participants first rated their negative emotions of the past week. Then, they completed a ∼50-min mini-computerized cognitive battery, including inhibitory control, cognitive flexibility, divided attention, and working memory. The experiment environment was a quiet room. A well-trained graduate student (MX) instructed and monitored tests of all participants. After the third test session, participants were tracked for their subsequent menses to validate their predicted cycle phase falling into the normal range. Those who violated were excluded from analysis even after completing the study.

### Self-Assessment Scales

#### National Aeronautics and Space Administration Task Load Index

The NASA-TLX measured the subjective workload using six subscales: mental demand, physical demand, temporal demand, performance, effort, and frustration levels (Tubbs-Cooley et al., 2018). Each subscale is rated on a 20-point scale (0 = low, 20 = high), but for the performance scale (0 = good, 20 = poor). Higher scores indicate increased workloads.

#### Depression Anxiety Stress Scale-21

The Depression Anxiety Stress Scale-21 (DASS-21) measured negative emotions in the past week, including depression, anxiety, and stress subscale. Each subscale contains seven items. Each item was scored on a 4-point scale ranging from 0 to 3. The final score was the summed score multiplied by two (Henry and Crawford, 2005). Higher scores indicate higher depression, anxiety, or stress levels, respectively.

### The Mini-Computerized Cognitive Battery

The mini-computerized cognitive battery was developed using GNU Octave and Psychtoolbox 3.16 (Brainard, 1997; Pelli, 1997) under the UBUNTU 18.04 system on a Thinkpad T61 laptop (12-inch, 1024 × 768 pixels, 50 Hz refresh rate). Participants completed the battery sitting about 50 cm in front of the laptop screen in a quiet room. The battery included four tasks measuring inhibitory control, cognitive flexibility, divided attention, and working memory capacity.

#### Measure of Inhibitory Control

**FIGURE 1:**
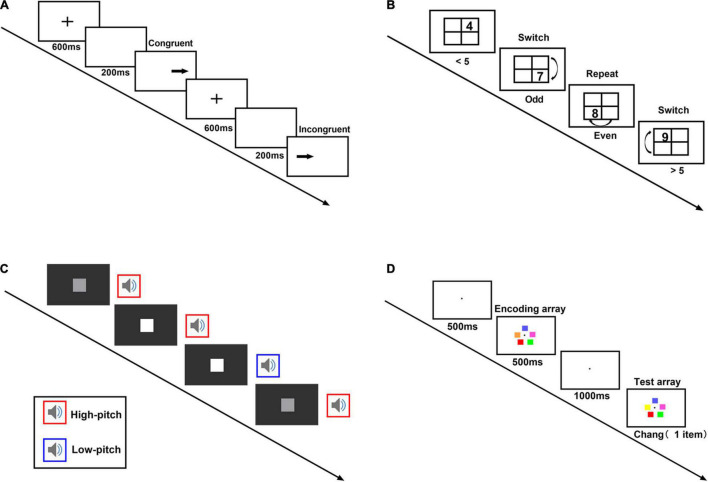
The mini-computerized cognitive battery. **(A)** Spatial Stroop task. A typical trial starts with a fixation (600 ms). After a blank screen (200 ms), an arrow appears on the left or the right side of the screen. Participants needed to report the arrow direction by pressing corresponding keys as soon as possible with the accuracy ensured. **(B)** Task-switch task. On each trial, a number (randomly selected from 1, 2, 3, 4, 6, 7, 8, 9) appeared in a cell of a 2 × 2 grid. If the number appeared in the upper row, participants needed to respond whether it was greater or less than 5. If the number appeared in the lower row, they answered whether it was even or odd. The first number appeared in the right-top cell and changed location clock-wisely in the subsequent trials. **(C)** Divided attention task. A square appears at regular intervals on the screen, and at the same time, participants listen to a sound. Every trial appeared every 1 s and lasted 1 s each. Participants needed to detect changes in either the visual sequence or the audio sequence. Whenever the square gets noticeably lighter, or the sound gets noticeably higher pitch twice in a row, they need to press the space bar as soon as possible. **(D)** Working memory task. A typical trial starts with a fixation (500 ms), followed by an encoding array (5 colored squares). The colored squares appeared on an imaginary circle (500 ms). A test array appeared after a 1,000 ms delay. The test array could have 0, 1, 2, or 5 changed items with equal probability. Participants had to indicate whether the test array and the encoding array differed.

The spatial Stroop task is a paradigm measuring inhibitory control (Aidman et al., 2019). A typical trial starts with a fixation in the screen center for 600 ms. After a blank screen of 200 ms, an arrow pointing leftward or rightward appeared on the left or the right side of the screen. Participants needed to report the arrow direction by pressing corresponding keys as soon as possible with accuracy ensured. The primary interest variable was the congruency between the arrow direction (leftward, rightward) and their position (left side, right side) (Figure 1A). There were 10 practice trials and 60 test trials, including 24 congruent and 36 incongruent trials. The error rate for each condition was summarized. We also calculated the mean reaction time after removal of error trials and trials too fast (< 150 ms) or too slow (> 1,500 ms).

#### Measure of Cognitive Flexibility

The task-switching paradigm measures cognitive flexibility (Monsell, 2003; Aidman et al., 2019). A number randomly chosen from 1 to 9 appeared in one cell of a 2 × 2 grid for each trial. If the number appeared in the upper row, participants needed to respond whether it was greater or less than 5. If the number appeared in the lower row, they answered whether it was even or odd. The first number appeared in the right-top cell and changed location clockwise in the subsequent trials. The trial was in repeat condition (same task rule) if the number appeared on the top-right and bottom-left cell; other trials were in switch condition (change of task rule) (Figure 1B). There were 12 practice trials and 60 test trials, including 29 repeat trials (with the first trial discarded) and 30 switching trials. We calculated error rates and mean reaction time for each condition. Error trials and trials too fast (< 150 ms) or low (> 1,500 ms) were excluded from reaction time analysis.

#### Measure of Divided Attention

The audiovisual cross-modal monitoring task measures divided attention ability (Himi et al., 2019). A square appears at regular intervals on the screen center, and, at the same time, participants listen to a sound. Participants needed to detect changes in either the visual sequence or the audio sequence. Sometimes the square gets noticeably lighter, and sometimes the sound gets a noticeably higher pitch. Whenever the square gets noticeably lighter or the sound gets noticeably higher pitch twice in a row, they need to press the space bar as soon as possible (Figure 1C). Thirty-two practice trials were followed by the test trials, which consisted of 200 trials. Every trial appeared every 1 s and lasted 1 s each. The index of the task was the sensitivity calculated according to signal detection theory using the non-parametric sensitivity measure (*A*′) (Stanislaw and Todorov, 1999) and the mean reaction time of correct responses.

#### Measure of Working Memory Capacity

The multiple change detection paradigm estimates working memory capacity (Gold et al., 2019). A typical trial starts with a fixation in the screen center for 500 ms, followed by an encoding array (5 colored squares). The colored squares appeared on an imaginary circle with a radius of 120 pixels centered on the center of the screen for 500 ms. A test array appeared after a 1,000-ms delay. The test array could have 0, 1, 2, or 5 changed items with equal probability. Participants had to indicate whether the test array and the encoding array differed (Figure 1D). There were 240 trials with 60 trials for each change type. The working memory capacity (*K*) was estimated according to a computational model (Feuerstahler et al., 2019) and used R (R Core Team, 2021) and the est_KAG function.^1^

### Statistical Analysis

Participants were classified into high and low workload groups using the mean value of NASA TLX total scores of all participants as the cut-off criterion. The group difference was examined using the independent-samples *t*-test and chi-square test. We conducted an omnibus mixed factorial analysis of variance (ANOVA) first for each emotion and task measure and performed *post hoc* comparisons using the LSD method if necessary. The *p*-value was adjusted using the Greenhouse–Geisser procedure in case of violation of the sphericity hypothesis. We translate *p*-values in the language of evidence to avoid the black-or-white null-hypothesis testing with an arbitrary *p*-value cut-off (Muff et al., 2022). The statistical analysis software was IBM SPSS Statistics for Windows (Version 22.0. Armonk, NY, United States: IBM). To exclude the practice effect and potential confounding effect of age, we also conducted linear mixed model analyses by controlling the effect of the session and participants’ age. The supplementary analysis, in the form of an *Rnotebook*, is available online (see Data Availability Statement). The linear mixed-effect model was conducted using *R (version 4.1.1)* (R Core Team, 2021), *afex* (Singmann et al., 2021), and *lme4* (Bates et al., 2015) package.

## Results

### Subjective Workload

We divided participants into low (*n* = 41) and high (*n* = 38) workload groups according to the mean NASA-TLX score (*M* = 69.65, SD = 14.81). Table 1 compares the low and high workloads groups on demographic information and mental health measures.

**TABLE 1:** Demographics and work characteristics of the sample.

Variables	Low (*n* = *41*)	High (*n* = *38*)	*p*-value
Cycle length, mean (SD)	29.6 (1.81)	29.2 (1.64)	0.224
Age, mean (SD)	24.1 (3.81)	27.1 (4.40)	0.002
BMI, mean (SD)	20.6 (2.65)	21.2 (2.07)	0.286
Marital status, *n* (%)			0.093
Single	32 (78.0%)	22 (57.9%)	
Married	9 (22.0%)	16 (42.1%)	
Education level, *n* (%)			0.098
Specialist qualification	29 (70.7%)	19 (50.0%)	
Bachelor degree	12 (29.3%)	19 (50.0%)	
Monthly income, *n* (%)			0.126
< 4000 RMB	30 (73.2%)	19 (50.0%)	
< 6000 RMB	9 (22.0%)	11 (28.9%)	
< 8000 RMB	1 (2.44%)	4 (10.5%)	
> 8000 RMB	1 (2.44%)	4 (10.5%)	
BDI, mean (SD)	3.05 (2.77)	2.66 (2.81)	0.536
GAD-7, mean (SD)	3.34 (2.52)	3.76 (2.89)	0.493

*BMI, Body mass index; BDI, Beck depression inventory; GAD-7, Generalized anxiety disorder 7-item scale; SD, Standard deviation.*

### Effect of Workload and Menstrual Cycle on Negative Emotion

A mixed factorial ANOVA of 2 (group: low, high workload) × 3 (cycle phase: menses, late follicular, mid-luteal) was conducted for depression, anxiety, and stress scores, respectively. The results revealed no evidence that the high and the low workload group differed on each emotion subscale (all *ps* > 0.29, see Supplementary Table 1 for detailed information). There was no evidence of the effect of the cycle phase, no matter in terms of main effect or interaction effect, on the depression and stress scores (all *ps* > 0.1). However, there was weak evidence of the main effect of the cycle phase on the anxiety score (*p* = 0.071).

### Effect of Workload and Menstrual Cycle on Inhibitory Control

A mixed factorial ANOVA of 2 (group: low, high workload) × 3 (cycle phase: menses, late follicular, mid-luteal) × 2 (congruency: congruent, incongruent) was conducted on error rate and reaction time, respectively. See Supplementary Table 2 for a summary of the ANOVA analysis.

On the measure of error rate, there was moderate evidence for a main effect of congruency [*F*(1, 77) = 4.873, *p* = 0.03, η*^2^* = 0.06], indicating generally more errors in the incongruent (*M* = 0.020, SE = 0.003) than the congruent condition (*M* = 0.014, SE = 0.002). The statistical evidence supporting the main effect of group and cycle phase was little or no (all *ps* > 0.2). Besides, we found little or no evidence for the two-way interactions (all *ps* > 0.05). However, there was moderate evidence for the interaction effect among the three factors [*F*(2, 154) = 3.885, *p* = 0.023, η*^2^* = 0.048]. A repeated-measures ANOVA of 3 (cycle phase: menses, late follicular, and mid-luteal) × 2 (congruency: congruent, incongruent) was then performed for the low workload group and the high workload group, respectively.

**FIGURE 2:**
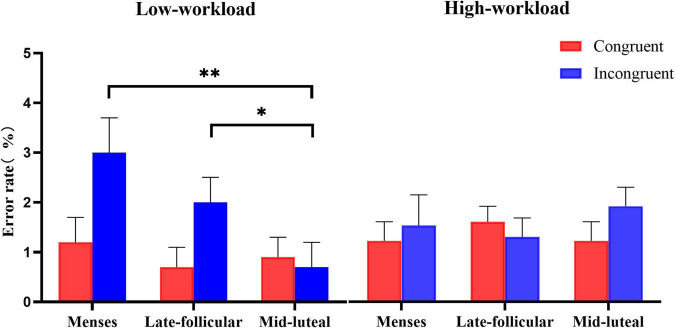
The interactive effect of workload and the menstrual cycle on the error rate measure of the spatial Stroop task. Error bars represent the standard error of the mean. * and ** indicates *p* < 0.05 and *p* < 0.01, correspondingly.

There was only weak evidence for the low workload group for the main effect of the cycle phase [*F*(1.56, 62.3) = 2.87, *p* = 0.076, η*^2^* = 0.067]. However, there was strong evidence for the main effect of congruency [*F*(1, 40) = 7.76, *p* = 0.008, η*^2^* = 0.163], and moderate evidence for the interaction effect between the cycle phase and the congruency [*F*(2, 80) = 4.56, *p* = 0.013, η*^2^* = 0.102]. Follow-up analyses found no evidence that performance on the congruent condition varied among the cycle phase [*F(*2, 39) = 0.39, *p* = 0.68, η*^2^* = 0.02]. However, on the error rate of the incongruent condition, there was strong evidence for the main effect of the cycle phase [*F(*2, 39) = 5.76, *p* = 0.006, η*^2^* = 0.23]. Error rates of the mid-luteal phase were lower than the menses (*p* = 0.003) and the late follicular phase (*p* = 0.037). There was little or no evidence for the difference between the menses and the late follicular phase on the incongruent condition (*p* = 0.183). We found little or no evidence for the main effect and interaction term involving the cycle phase in the high workload group [cycle: *F*(2, 74) = 0.17, *p* = 0.846, η*^2^* = 0.005, cycle × congruency: *F*(2, 74) = 1.18, *p* = 0.312, η*^2^* = 0.031]. See Figure 2 for an illustration.

On the measure of reaction time, there was strong evidence for the main effect of congruency [*F*(1, 77) = 12.759, *p* = 0.001, η*^2^* = 0.142], indicating faster responses in the congruent condition (*M* = 487 ms, SE = 8 ms) than the incongruent condition (*M* = 499 ms, SE = 8 ms). We also found moderate evidence for the main effect of group [*F*(1, 77) = 5.353, *p* = 0.023, η*^2^* = 0.065], suggesting that the high workload group (*M* = 476 ms, SD = 11 ms) responded faster than the low workload group (*M* = 511 ms, SD = 11 ms) in general. However, we found little or no evidence for the main effect or interaction effect involving cycle phase (all *ps* > 0.05).

### Effect of Workload and Menstrual Cycle on Cognitive Flexibility

A mixed 2 (group: low, high workload) × 3 (cycle phase: menses, late follicular, mid-luteal) × 2 (condition: repeat, switch) factorial ANOVA was conducted on error rate and reaction time, respectively. See Supplementary Table 3 for a summary of the ANOVA analysis.

**FIGURE 3:**
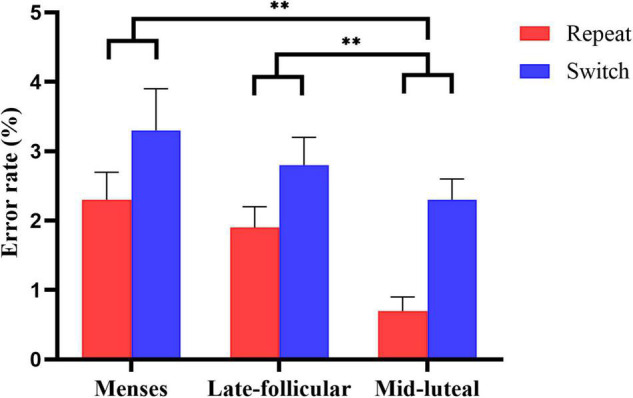
The main effect of the menstrual cycle on the error rate measure of the task-switching task. Error bars represent the standard error of the mean. ** indicates *p* < 0.01.

On the measure of error rate, there was very strong evidence for the main effect of condition [*F*(1, 77) = 18.058, *p* < 0.001, η*^2^* = 0.19], suggesting more errors in the switch condition (*M* = 0.028, SE = 0.003) than the repeat condition (*M* = 0.016, SE = 0.002). We also found strong evidence for the main effect of cycle phase [*F*(1.703, 131.121) = 5.271, *p* = 0.009, η*^2^* = 0.064], indicating fewer errors in the mid-luteal phase than the menses phase (*p* = 0.006) and late follicular phase (*p* = 0.007). However, there was little or no evidence that the menses and the late follicular phase differ (*p* = 0.317). See Figure 3 for an illustration. The results revealed little or no evidence for the main effect of group [*F*(1, 77) = 0.628, *p* = 0.431, η*^2^* = 0.008]. In addition, we found little or no evidence for the two-way and three-way interactions (all *ps* > 0.2, see Supplementary Table 3).

On the measure of reaction time, there was a strong evidence for the main effect of condition [*F*(1, 77) = 445.941, *p* < 0.001, η*^2^* = 0.853], indicating slower responses in the switch (*M* = 987 ms, SE = 15 ms) than the repeat condition (*M* = 825 ms, SE = 15 ms). However, we found little or no evidence for the main effect of the group and cycle phase and the two-way and three-way interactions (all *ps* > 0.45, see Supplementary Table 3).

### Effect of Workload and Menstrual Cycle on Divided Attention

A mixed 2 (group: low, high workload) × 3 (cycle phase: menses, late follicular, mid-luteal) factorial ANOVA was conducted on the sensitivity measure (*A*′) and reaction time, respectively. See Supplementary Table 4 for a summary of the ANOVA analysis.

**FIGURE 4:**
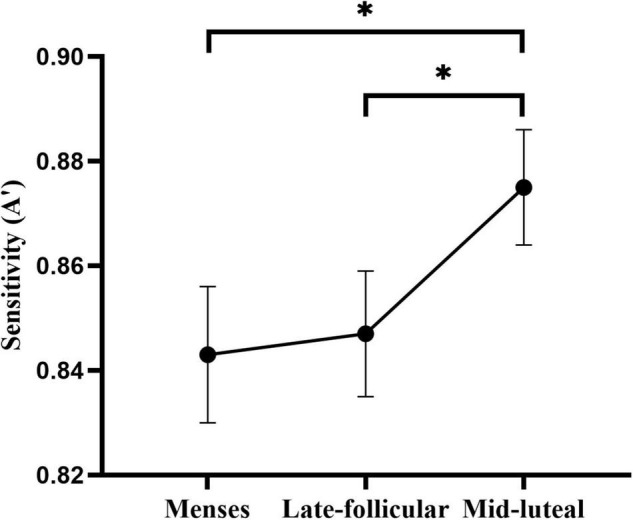
The main effect of the menstrual cycle on the sensitivity measure of the divided attention task. * indicates *p* < 0.05.

The ANOVA on the sensitivity measure (*A*′) revealed moderate evidence for the main effect of cycle phase [*F*(2, 154) = 3.29, *p* = 0.035, η*^2^* = 0.042]. *Post hoc* analysis indicated higher *A’* in the mid-luteal phase than in the menses phase (*p* = 0.021) and late follicular phase (*p* = 0.030). However, there was no evidence of the difference between the menses and the late follicular phase (*p* = 0.780) (Figure 4). We also found little or no evidence for the main effect and interaction terms involving the group (all *ps* > 0.17, see Supplementary Table 4).

**FIGURE 5:**
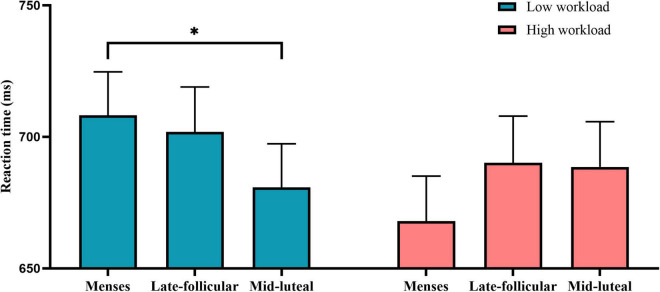
The interactive effect of workload and the menstrual cycle on the reaction time measure of the divided attention task. Error bars represent the standard error of the mean. * indicates *p* < 0.05.

On the measure of reaction time, there was little or no evidence for the main effect of cycle phase [*F*(2, 154) = 0.74, *p* = 0.479, η*^2^* = 0.01] or group [*F*(1, 77) = 0.473, *p* = 0.494, η*^2^* = 0.006]. However, the results revealed moderate evidence for the interaction between the group and cycle phase [*F*(2, 154) = 3.21, *p* = 0.043, η*^2^* = 0.04]. Follow-up analyses revealed that moderate evidence that participants of low workload responded faster during the mid-luteal phase than the menses (*p* = 0.037) and little or no evidence for other comparisons (mid-L vs. late-F: *p* = 0.12; late-F vs. menses: *p* = 0.635). However, there was no evidence for the cycle effect in the high workload group (all *ps* > 0.11) (Figure 5).

### Effect of Workload and Menstrual Cycle on Working Memory

A mixed 2 (group: low, high workload) × 3 (cycle phase: menses, late follicular, mid-luteal) factorial ANOVA on the working memory capacity estimation (*K*) was conducted. See Supplementary Table 5 for a summary of ANOVA results. However, there was no evidence for the main effects [group: *F*(1, 77) = 0.563, *p* = 0.455, η*^2^* = 0.007; cycle phase: *F*(2, 154) = 0.705, *p* = 0.496, η*^2^* = 0.009] and the interaction effect [*F*(2, 154) = 0.009, *p* = 0.991, η*^2^* < 0.001]. See Supplementary Table 5 for detailed information.

## Discussion

The present study investigates cognitive performance across the menstrual cycle using a sample of nurses with similar duties. As summarized in Table 2, our results demonstrate evidence for the general cognitive advantage of the mid-luteal phase, as manifested by the main effect of the menstrual cycle on the error rate measure of the task-switch task and the sensitivity measure of the divided attention task. Moreover, the present study demonstrates that workload might be a modulatory factor of the menstrual cycle effect, with preliminary evidence that the cycle phase effect on the reaction time measure of divided attention task and the error rate measure of response inhibition task is only manifested in the low workload group.

**TABLE 2:** Summary of main findings involving menstrual cycle.

Task	Measure	Cycle	Cycle × Workload	Cycle × Workload × Condition
Inhibitory control	Error rate	0.274	0.096	**0.023**
	RT	0.224	0.660	0.066
Cognitive flexibility	Error rate	**0.009**	0.701	0.876
	RT	0.531	0.599	0.498
Divided attention	Sensitivity	**0.035**	0.224	–
	RT	0.479	**0.043**	–
Working memory	Capacity	0.496	0.991	–

*RT, Reaction time; The Cycle × Workload × Condition is not available as no experimental contrast was defined; The values in the table refer to p-values and are shown in bold when p < 0.05.*

### The Main Effect of the Menstrual Cycle

Our results demonstrate a mid-luteal phase advantage on the error rate measure of task-switching. The task-switching paradigm adopted in the current research is a classical measure of cognitive flexibility (Monsell, 2003). Our results replicate a typical switch-cost phenomenon: people make slower responses and more errors when the task rule is switched compared with repeat. Using the Wisconsin Card Sorting Task, Solis-Ortiz et al. (2004) reported a similar finding that females show superior cognitive flexibility during their luteal phase. A recent study using a similar task-switching task in functional MRI (fMRI) demonstrated that enhanced prefrontal activations after hormone therapy (sequential estradiol-plus-progesterone) were associated with improved task-switching performance in a sample of early menopausal women (Girard et al., 2017). However, Girard et al. (2017) did not observe beneficial effects on behavioral measures. Their failure to detect behavioral effects might be due to the task design tailored for fMRI or the small sample size.

It should be noteworthy that we found no evidence for the interaction between the menstrual cycle and the switch condition. In other words, the menstrual cycle modulates the general task performance, but not the switching cost in our study. Thus, the performance improvement in the mid-luteal phase might not specifically suggest the ovarian hormone’s effect on the task-switching process. The general task performance improvement in the task-switch task is likely due to an attentional augment mechanism. Female participants in our study might show relatively good skills in neglecting task-irrelevant and focusing on task-relevant information during their mid-luteal phase. This hypothesis is consistent with our finding on the divided attention task.

On the divided attention task, female nurses in our research show superior sensitivity in detecting the visual and auditory changes during their mid-luteal phase. We adopted a cross-modal monitoring task to assess participants’ ability to simultaneously attend to visual and auditory modalities (Himi et al., 2019). The effect of hormones or the menstrual cycle on different facets of attention has been explored, such as sustained attention (Solis-Ortiz and Corsi-Cabrera, 2008), selective attention (Thimm et al., 2014; Brotzner et al., 2015; Wang and Chen, 2020), and divided attention (Leeners et al., 2017; Pletzer et al., 2017). A seminal work from Pletzer et al. (2017) systematically examined the sex and menstrual cycle effect on three aspects of attention, which reports a follicular phase advantage on the accuracy measure of divided and sustained attention. Unlike us, Pletzer et al. (2017) use paper-pencil tests to assess selective and divided attention. Moreover, Pletzer et al. (2017) only compared the luteal and follicular phases, making a direct comparison with us impossible. Leeners et al. (2017) adopted a similar bimodal attention task as us, but they failed to detect any association between hormone levels and divided attention (Leeners et al., 2017). However, Leeners et al. (2017) used a very heterogeneous sample, including both endocrine disorders and healthy females, making a direct comparison with us impossible.

The general cognitive advantage of the mid-luteal phase, manifested in the task switch and divided attention task, is consistent with recent evidence of the progesterone effect on prefrontal function (Dubol et al., 2021). The prefrontal cortex plays essential roles in cognitive control, influencing attention, impulse inhibition, prospective memory, and cognitive flexibility. A recent systematic review of multimodal neuroimaging studies suggests that enhanced prefrontal activations in the middle luteal phase are a convergent finding in the literature (Dubol et al., 2021). For example, Pletzer et al. (2019) investigated brain activations and functional connectivity changes when women perform a spatial navigation task and a verbal fluency task during the menstrual cycle. Intriguingly their study reveals that progesterone increases the BOLD responses of the dorsal prefrontal cortex and caudate during the luteal cycle phase irrespective of the task (Pletzer et al., 2019). Whether the main effect of the menstrual cycle on task switching and divided attention performance was driven by progesterone’s impact on the prefrontal cortex requires additional research efforts. Future studies might use fMRI to clarify this issue.

### Workload as a Modulatory Factor

This study reveals intriguing interactions between the error rate measure of the spatial Stroop task and the reaction time measure of the divided attention task. Analysis of the two tasks reveals a similar finding that only low workload groups performed better during their mid-luteal phases. However, the mid-luteal cognitive advantage disappeared in high workload groups.

In the spatial Stroop task, participants make a speeded response to the arrow direction and inhibit the dominant tendency to respond with the ipsilateral hand matching the arrow position when direction and position information conflict. Cognitive control is necessary to focus on the task-relevant information (selective attention) and inhibit the dominant response tendency (response inhibition) (Pires et al., 2018). Unlike us, previous studies mainly used the color Stroop task, and the results were inconsistent (Hatta and Nagaya, 2009; Hidalgo-Lopez and Pletzer, 2017). For example, a study reported that females performed worse during their luteal phase than during the menses phase (Hatta and Nagaya, 2009). In contrast, we did not find evidence for the main effect but evidence for the interactive effect of the menstrual cycle. There was also moderate evidence for the interaction effect on the reaction time measure of the divided attention task.

Our findings parallel recent studies on modulatory factors (Jacobs and D’Esposito, 2011; Hidalgo-Lopez and Pletzer, 2017, 2019; Bernal and Paolieri, 2022). A recent study demonstrates that the menstrual cycle effect on color Stroop task performance is modulated by the baseline dopamine levels (Hidalgo-Lopez and Pletzer, 2017). Their following research used the stop-signal fMRI task to measure inhibitory control and associated brain activity, indicating the baseline inhibitory control might also be a potential modulating variable (Hidalgo-Lopez and Pletzer, 2019). In addition, the recent review proposes that it is crucial to consider modulating factors to avoid confounding findings (Bernal and Paolieri, 2022). Those pieces of evidence, along with us, advocate research attention to potential modulating factors that might change the direction or strength of the menstrual cycle effect.

It is noteworthy that the mid-luteal phase advantage was eliminated in the high workload group on both the error rate measure of the spatial Stroop task and the reaction time measure of the divided attention task. These preliminary findings imply that work-related stress might offset the protective effect of ovarian steroids. Previous studies have suggested that hormones from the HPA axis are involved in regulating the HPG axis at different levels (Oyola and Handa, 2017). The HPA axis is the coordinator of the brain’s fight-or-flight response, which increases cortisol production to deal with stressful events. A recent study indicates that the hair cortisol concertation predicts work-related stress only in the high workload condition but not in the normal workload condition (van der Meij et al., 2018). The increased perceived workload in the high workload group did not affect emotion yet as we did not find the workload effect on the DASS scores. We failed to detect the workload effect on emotion, but “cold” prefrontal-mediated tasks might be due to the rating scale’s insensitivity. Another explanation is a complex interaction among the HPA axis, HPG axis, and the prefrontal network underneath women’s cycling cognitive and affective performance. Our preliminary findings advocate future research efforts to tease apart the potential dynamics among the “cold”/“hot” brain systems and the neuroendocrine system.

Contrary to our expectations, we did not find an effect of workload and menstrual cycle on working memory. Visual working memory is essential for cognitive performance (Luck and Vogel, 2013). The present study estimates participants’ visual working memory capacity (*K*) for each test session using a multiple-change detection paradigm (Gold et al., 2019) and a computational model (Feuerstahler et al., 2019). However, this study did not reveal the workload or the menstrual cycle’s effect on the *K* index, consistent with a recent study using a single probe change detection paradigm (Wassell et al., 2015). Although Wassell et al. (2015) revealed that progesterone levels in the mid-luteal phase modulate mental imagery ability, but they failed to find any association between cycle phase, hormone concentration, and working memory performance.

Previous studies on the menstrual cycle effect primarily used verbal working memory tasks (Joseph et al., 2012; Hidalgo-Lopez and Pletzer, 2021). A recent study found enhanced frontal activity and disinhibition of the salience brain network and striatum in a verbal working memory task (letter N-back) during the luteal phase (Hidalgo-Lopez and Pletzer, 2021). Hampson and Morley (2013) suggest that estradiol, but not progesterone levels, is associated with spatial working memory performance using a sample of women of reproductive age. Their study implies that females might perform best during their follicular phase when the estradiol levels are high. However, using a working memory task for emotional expressions, Gasbarri et al. (2008) indicated that working memory is impaired in the follicular phase.

The inconsistent findings in the literature might be due to methodological differences. Another potential explanation might be complex interactions among the HPA axis, HPG axis, and neurotransmitter systems, such as the dopaminergic system. Previous studies have suggested an inverted U-shape relationship between dopamine concentration and prefrontal cortex mediated cognitive function, such as working memory and cognitive control (Cools and D’Esposito, 2011). Recent studies have found that the mid-luteal phase and progesterone levels drive the effects of dopamine and cycle interactions on cognitive control (Hidalgo-Lopez and Pletzer, 2017). The picture gets increasingly complex by considering another inverted U-shape association between workload and task performance (Ma et al., 2020). It is possible that complex interactions among progesterone, dopamine, and workload obscure the findings of this study. Alternatively, it may be due to other mechanisms, such as functional compensation in the brain. Although this study provides insights on potential intriguing modulating mechanisms, clarifying the exact mechanism is far from our reach. Increasing research efforts are necessary for the future.

### Limitations and Future Directions

This study used a validated backward-counting procedure to determine the late-follicular and mid-luteal phases. To further minimize the impact of menstrual cycle mapping error, we increase the sample sizes. As far as we know, few studies have a sample size bigger than us (*n* = 79) if they used the longitudinal design with a homogenous sample like us. In addition, we double-checked and excluded participants if their actual menses onset deviated from the normal range during the experiment. Despite this, we admit that it might comprise a potential limitation without saliva, urine, or blood test to verify the hormone levels.

Although our results indicate workload as a modulatory factor on the menstrual cycle’s effect on cognition, caution should be made that the evidence is preliminary. Future research is still necessary to replicate the role of workload with samples of females in other workplace settings. In addition, the workload is a too complex construct that might confound many other concepts. Moreover, participants in the present study rated their generally experienced workload in the past 3 months, not their workload at the moment. Thus, the current findings might not answer how acute work stress impacts the menstrual cycling effect. Future studies might use new research methodology, such as experience sampling (Bos et al., 2015) and wearable neurophysiological recordings (Yokota et al., 2017), to provide an objective and immediate measure of workload.

This study contributes a mini-computerized cognitive battery specifically designed to evaluate four cognitive skills critical for nursing performance. We make it publicly available to make replicative and collaborative research works possible. However, we need to emphasize that the tasks in the battery are only a tiny subset of cognitive assessment and may not capture the cognitive performance at work. We suggest that it is valuable to assess cognitive performance by tracking operational errors when nurses perform routine tasks in their workplace.

## Conclusion

How the menstrual cycle impacts the cognitive performance of females in the workplace is less understood. The present study employed a sample of nurses with similar duties and tracked their cognitive performance during their menses, late-follicular, and mid-luteal phases. Our results demonstrate a general mid-luteal advantage in error rate measure of task-switching and sensitivity measure of divided attention. Moreover, the present study reveals preliminary evidence that workload modulates the menstrual cycle effect on cognition. Only females with low workload manifest the mid-luteal cognitive advantage on the reaction time measure of divided attention and the error rate measure of response inhibition, implying that a suitable workload threshold might be necessary for regular neuro-steroid interactions. Thus, this study advocates the significance of research focused on the brain cycle under workloads.

## Data Availability Statement

The datasets presented in this study can be found in online repositories. The names of the repository/repositories and accession number(s) can be found below: https://www.doi.org/10.11922/sciencedb.o00068.00001.

## Ethics Statement

The studies involving human participants were reviewed and approved by the Hefei Cancer Hospital, Chinese Academy of Sciences. The patients/participants provided their written informed consent to participate in this study.

## Author Contributions

L-ZY, MX, HW, and HL designed and made the concept of the study and performed the analysis and interpretation of the data. L-ZY, HW, and HL contributed research tools. MX and DC were responsible for data acquisition. MX and L-ZY drafted the manuscript, which was critically revised and approved by all authors. All authors agreed to be accountable for all aspects of the work in ensuring that questions related to the accuracy or integrity of any part of the work are appropriately investigated and resolved.

## Conflict of Interest

The authors declare that the research was conducted in the absence of any commercial or financial relationships that could be construed as a potential conflict of interest.

## Publisher’s Note

All claims expressed in this article are solely those of the authors and do not necessarily represent those of their affiliated organizations, or those of the publisher, the editors and the reviewers. Any product that may be evaluated in this article, or claim that may be made by its manufacturer, is not guaranteed or endorsed by the publisher.

## Footnotes

1
https://github.com/leahfeuerstahler/vwm
