# Supplementary material for: The Cycling Brain in the Workplace: Does Workload Modulate the Menstrual Cycle Effect on Cognition?
Source: Front Behav Neurosci. 2022 Jun 2;16:856276. doi: 10.3389/fnbeh.2022.856276 (PMC9201761; doi:10.3389/fnbeh.2022.856276)
Supplement: Supplementary file 1 [file Data_Sheet_1.docx]

**Supplementary material** **for**

**The Cycling Brain in the Workplace: Does Workload Modulate the** **Menstrual Cycle Effect on Cognition?**

**Min Xu^1,2,3^, Dandan Chen^2^, Hai Li^2,3^, Hongzhi Wang^1,2,3^*, Li-Zhuang Yang^2,3*^**

^1^ Bengbu Medical College, Bengbu, China

^2^ Hefei Cancer Hospital, Chinese Academy of Sciences, Hefei, China

^3^ Anhui Province Key Laboratory of Medical Physics and Technology, Institute of Health and Medical Technology, Hefei Institutes of Physical Science, Chinese Academy of Sciences, Hefei, China

***Table S1****: The ANOVA results of negative emotions.*

|  | Predictor | *df_Num_* | *df_Den_* | *F* | *p* | η^2^_p_ |
| --- | --- | --- | --- | --- | --- | --- |
| Depression |  |  |  |  |  |  |
|  | group | 1 | 77 | 0.001 | 0.981 | 0.000 |
|  | cycle | 2 | 154 | 2.175 | 0.12 | 0.027 |
|  | group x cycle | 2 | 154 | 0.802 | 0.45 | 0.01 |
|  |  |  |  |  |  |  |
| Anxiety |  |  |  |  |  |  |
|  | group | 1 | 77 | 0.001 | 0.978 | 0.000 |
|  | cycle | 1.843 | 141.9 | 2.769 | 0.071 | 0.035 |
|  | group x cycle | 1.843 | 141.9 | 0.274 | 0.743 | 0.004 |
|  |  |  |  |  |  |  |
| Stress |  |  |  |  |  |  |
|  | group | 1 | 77 | 1.126 | 0.292 | 0.014 |
|  | cycle | 2 | 154 | 0.089 | 0.915 | 0.001 |
|  | group x cycle | 2 | 154 | 0.191 | 0.827 | 0.002 |

*Note.* *df_Num_* indicates degrees of freedom numerator. *df_Den_* indicates degrees of freedom denominator. η^2^_p_ indicates partial eta-square.

***Table S2****: The ANOVA results of inhibitory control.*

|  | Predictor | *df_Num_* | *df_Den_* | *F* | *p* | η^2^_p_ | |
| --- | --- | --- | --- | --- | --- | --- | --- |
| Error rate |  |  |  |  |  | |  |
|  | group | 1 | 77 | 0.978 | .326 | | .013 |
|  | congruency | 1 | 77 | 4.873 | **.03** | | .06 |
|  | group x congruency | 1 | 77 | 1.485 | .227 | | .019 |
|  | cycle | 1.802 | 138.724 | 1.3 | .274 | | .017 |
|  | group x cycle | 1.802 | 138.724 | 2.441 | .096 | | .031 |
|  | cycle x congruency | 2 | 154 | 1.296 | .277 | | .017 |
|  | group x cycle x congruency | 2 | 154 | 3.885 | **.023** | | .048 |
|  |  |  |  |  |  | |  |
| Reaction time |  |  |  |  |  | |  |
|  | group | 1 | 77 | 5.353 | **.023** | | .065 |
|  | congruency | 1 | 77 | 12.759 | **.001** | | .142 |
|  | group x congruency | 1 | 77 | 2.181 | .144 | | .028 |
|  | cycle | 1.576 | 121.347 | 1.526 | .224 | | .019 |
|  | group x cycle | 1.576 | 121.347 | 0.341 | .660 | | .004 |
|  | cycle x congruency | 1.778 | 136.881 | 0.122 | .863 | | .002 |
|  | group x cycle x congruency | 1.778 | 136.881 | 2.87 | .066 | | .036 |

*Note.* *df_Num_* indicates degrees of freedom numerator. *df_Den_* indicates degrees of freedom denominator. η^2^_p_ indicates partial eta-square.

***Table S3****: The ANOVA results of cognitive flexibility.*

|  | Predictor | *df_Num_* | *df_Den_* | *F* | *p* | η^2^_p_ |
| --- | --- | --- | --- | --- | --- | --- |
| Error rate |  |  |  |  |  |  |
|  | group | 1 | 77 | 0.628 | .431 | .008 |
|  | condition | 1 | 77 | 18.058 | **.0001** | .19 |
|  | group x condition | 1 | 77 | 1.184 | .280 | .015 |
|  | cycle | 1.703 | 131.121 | 5.271 | **.009** | .064 |
|  | group x cycle | 1.703 | 131.121 | 0.307 | .701 | .004 |
|  | cycle x condition | 2 | 154 | 0.7 | .498 | .009 |
|  | group x cycle x condition | 2 | 154 | 0.132 | .876 | .002 |
|  |  |  |  |  |  |  |
| Reaction time |  |  |  |  |  |  |
|  | group | 1 | 77 | 0.389 | .535 | .005 |
|  | condition | 1 | 77 | 445.941 | **.000** | .853 |
|  | group x condition | 1 | 77 | 0.013 | .909 | .000 |
|  | cycle | 2 | 154 | 0.636 | .531 | .008 |
|  | group x cycle | 2 | 154 | 0.515 | .599 | .007 |
|  | cycle x condition | 2 | 154 | 0.322 | .726 | .004 |
|  | group x cycle x condition | 2 | 154 | 0.70 | .498 | .009 |

*Note.* *df_Num_* indicates degrees of freedom numerator. *df_Den_* indicates degrees of freedom denominator. η^2^_p_ indicates partial eta-square.

***Table S4****: The ANOVA results of divided attention.*

|  | Predictor | *df_Num_* | *df_Den_* | *F* | *p* | η^2^_p_ |
| --- | --- | --- | --- | --- | --- | --- |
| Sensitivity |  |  |  |  |  |  |
|  | group | 1 | 77 | 1.891 | .173 | .024 |
|  | cycle | 2 | 154 | 3.29 | **.035** | .042 |
|  | group x cycle | 2 | 154 | 1.511 | .224 | .019 |
|  |  |  |  |  |  |  |
| Reaction time |  |  |  |  |  |  |
|  | group | 1 | 77 | 0.473 | .494 | .006 |
|  | cycle | 2 | 154 | 0.74 | .479 | .01 |
|  | group x cycle | 2 | 154 | 3.213 | **.043** | .04 |

*Note.* *df_Num_* indicates degrees of freedom numerator. *df_Den_* indicates degrees of freedom denominator. η^2^_p_ indicates partial eta-square.

***Table S5****: The ANOVA results of working memory.*

| Predictor | *df_Num_* | *df_Den_* | *F* | *p* | η^2^_p_ |
| --- | --- | --- | --- | --- | --- |
| group | 1.00 | 77.00 | 0.563 | .455 | .007 |
| cycle | 2 | 154 | 0.705 | .496 | .009 |
| group x cycle | 2 | 154 | 0.009 | .991 | .000 |

*Note.* *df_Num_* indicates degrees of freedom numerator. *df_Den_* indicates degrees of freedom denominator. η^2^_p_ indicates partial eta-square.
